# Cross Protectivity Analysis of 49.8 kDa Pili Subunits of *S. flexneri* against *Vibrio cholerae* Infection

**DOI:** 10.1155/2022/3751521

**Published:** 2022-06-15

**Authors:** Dwi Yuni Nur Hidayati, Septha Rully, Aisyah Amalia, Elsa Larissa Widyani, Genitri Indraswari, Adrian Prasetya, Merika Soraya, Sri Winarsih, Sumarno Reto Prawiro

**Affiliations:** ^1^Department of Clinical Microbiology, Faculty of Medicine, University of Brawijaya, Malang, Indonesia; ^2^Master Program in Biomedical Science, Faculty of Medicine, University of Brawijaya, Malang, Indonesia

## Abstract

**Background:**

Although the AMV and AMS vaccine candidates have similar characteristics as hemagglutinin and adhesive molecules, there are differences in molecular weight.

**Objective:**

The research aims to determine the immunological cross-reaction between AMS and AMV.

**Method:**

Antihemagglutination test used the anti-adhesion molecular antibody AMS. Next, we examined the immune response that has to be linked with protectivity. The model of the research uses MLIL. The sample separated the mice into four groups, and each group had five mice. The first group was the negative control group. The second group was given AMV and infected with *Shigella flexneri*. The third group was immunized with AMV before being exposed to *Shigella flexneri*. The last group was infected with *Vibrio cholerae*. The immune response results were evaluated by calculating the weight of MLIL and counting the colony of bacteria. We also examined other AMS immune responses, namely, *β*-defensin and s-IgA levels. To get the data, we measured the number of Th17 immune effector cells, T-reg, and proinflammatory cytokine IL-17A. Data analysis was performed using ANOVA, independent *t*-test, Kruskal–Wallis, and Mann–Whitney tests.

**Results:**

An antihemagglutination cross immune response, intestinal weight, the number of bacterial colonies, and other findings were found to be significant (*p* < 0.05) for the levels of *β*-defensin, s-IgA, Th17, T-reg, and IL-17A.

**Conclusion:**

The 49.8 kDa·MW protein subunit of the *Shigella flexneri* adhesion molecule could act as a candidate vaccine homologous for shigellosis and cholera in the future.

## 1. Introduction

Diarrhoea is a formidable foe in a developing country with poor sanitation and a lack of clean drinking water sources. Many kinds of microorganisms may cause diarrhoea. Some induce severe clinical manifestations, leading to death, such as cholera and shigellosis. Cholera is an acute diarrheal disease caused by the ingestion of food or water contaminated with *Vibrio cholerae* (*V. cholerae*). Therefore, cholera may kill an individual within hours if it remains untreated due to rapid dehydration. Researchers have estimated 1.3 million–4.0 million cholera cases with 21,000–143,000 death occurring annually in the endemic country [1].

Besides cholera, shigellosis is a significant cause of morbidity and mortality caused by diarrhoea. 1.88 million cases of shigellosis occur annually worldwide, with 164,000 associated death [2]. In a developing country, *Shigella* is the most common cause of bloody diarrhoea (dysentery) among children under five years and the second most common cause of diarrhoea overall [3].

The new intervention is necessary, including vaccine development, due to the high burden of cholera and shigellosis and multiple antibiotic resistance. Unfortunately, up until now, effective vaccines for cholera or shigellosis have been unsatisfactory. Cholera vaccines used nowadays include lyophilized CVD 103*-*HgR, Dukoral (manufactured by SBL vaccines), and ShanChol (produced by Shantha Biotech, India). Two vaccines based on live-attenuated or inactivated whole cells sometimes contain PAMP, which may cause side effects. The use of shigellosis vaccines based on O antigens was not accepted. The reason is due to various O polysaccharide antigens present in each species. So it is still necessary to develop a shigellosis vaccine candidate that can protect against each strain of *Shigella*.

In general, the path of bacteria in causing the disease consists of two stages. The first stage begins with the attachment of bacteria performed by the pili; the extension is the anchoring and then continues through the OMP, the docking. After the attachment of bacteria occurs, the bacteria will conduct colonization. During colonization, bacteria produce metabolic material. Some metabolites products endanger the host on pathogenic bacteria. These pathogenic bacterial groups include *S. flexneri* and *V. cholerae* [4].

Therefore, interfering with the initial bacterial attachment step will prevent bacteria from releasing devastating metabolic factors. Recent studies have identified AMV. The MW has 37.8 kDa as a part of pili bacteria. The AMS has MW 18 kDa, 23 kDa, 34 kDa, 49.8 kDa, and 7.9 kDa molecular weight in pili and 23 kDa and 27 kDa molecular weight in OMP [5].

The studies have known that there is cross-reactivity among *Shigella* sp. adhesion molecules such as cross-reactivity within 34 kDa subunit OMP adhesion molecule of *S. flexneri* with other *Shigella* species and then cross-reactivity within AMS with subunit 7.9 kDa in *S. flexneri* and *S. sonnei* [6]. Studies on the animal model have confirmed that vaccination using adhesion molecules of *Shigella dysenteriae* or *Vibrio cholerae* protects against intestinal infection by restricting bacterial colony growth and excessive intestinal fluid excretion [7, 8].

Our recent study has found that the hemagglutination and antihemagglutination tests showed that the antibody AMV could inhibit AMS and decrease intestinal weight and colony count of the vaccinated group compared to the naive group supporting cross-reaction. Vaccination also generates a higher Th17, T-reg, IL-17A, *β*-defensin, and s-IgA significantly [9].

We conducted this research to strengthen our previous study to clarify whether cross-reactivity occurs between AMS and AMV.

## 2. Materials and Methods

This study used an explorative and true experimental design research laboratory. Isolation of AMS, AMV, and anti-AMS antibodies was carried out exploratively in the first stage. Subsequently, in empirical laboratory design research, there were three steps. The first step was an antihemagglutination test for AMV, which used an anti-AMS antibody. The second step is to find out the immune response of the protectivity. This step used MLIL with mice Balb/c. After that, 20 Balb/c's male mice as the sample were divided into four groups. The first group was the negative control group. The second group was infected with *S. flexneri*. The third group was immunized with AMV before being affected by *S. flexneri*. The last group is the same as the third but infected with *V. cholerae*. The immune response of the protectivity calculates the weight and counts the bacterial colonies. The previous step finds out the others' reactions to Immun AMS, which calculated *β*-defensin and s-IgA levels. The cellular immune response measured the number of Th17 and T-reg immune effector cells and proinflammatory cytokines IL-17A.

### 2.1. The Explorative Study

#### 2.1.1. Culture of *S*. *flexneri* and *V*. *cholerae* and Isolation of Pili Protein

We obtained the sample bacteria from the Microbiology Laboratory of Faculty of Medicine Brawijaya Malang TCG (0.02% thioproline, 0.3% NaHCO3, 0.1% monosodium l-glutamate, 1% bactotryptone, 0.2% yeast extract, 0.5% NaCl, 2% bacto agar, and 1 mM *β*-aminoethyl ether-N,N,N',N'-tetraacetic acid (EGTA)). The TCG media were used to enhance pili production. 10 ml of bacteria was added to the media and incubated for 2 × 24 hours. The bacteria were collected and suspended with trichloroacetic acid until they reached 3% in concentration. The suspension was mixed using vortex for 30 s, stored at room temperature for 1 hour, and then centrifuged at 6000 rpm for 30 minutes at a temperature of 4°C. The centrifuged pellet was then resuspended with PBS pH 7.4 and pili isolation by using a bacterial pili cutter. Pili isolation is done with a bacterial pili cutter. Pili bacteria were sliced from the body of bacteria for six cycles. Each cycle of bacterial samples was centrifuged at 6000 rpm for 30 minutes at 4°C. The supernatant from each cutting, rich in pili fraction, is accommodated in an Eppendorf tube. After the last cycle, the supernatant was centrifuged at 12000 rpm for 15 min at 4°C, and then the supernatant-containing pili protein was placed in the Eppendorf tube and stored at −20°C [10].

#### 2.1.2. Sodium Dodecyl Sulfate-Polyacrylamide Electrophoresis (SDS-PAGE)

We measured the pili's molecular weight protein of *S. flexneri* and *V. cholerae*. According to Laemmli's method (1970), the SDS-PAGE evaluated the profile protein. The protein sample was heated at 100°C for 5 min in a buffer solution containing 5 mM Tris pH 6.8, 5% 2-mercaptoethanol, 2.5% w/v sodium dodecyl sulfate, and 10% v/v glycerol tracking gel 4%. Bromophenol was used as a colour tracker. The electrical voltage applied was 120 mV. We have done the coomassie brilliant blue as gel staining and the sigma standard low range molecular marker. The desired protein was then multiplied after measuring molecular weight [10].

#### 2.1.3. Pili Protein Purification of *V*. *cholerae* with Molecular Weight 37.8 kDa and *S*. *flexneri* with Molecular Weight 49.8 kDa

The protein interest was cut from electrophoresis gel at the protein. The method perpendicularly referred to the previous study. After that, we collected the cut band into a membrane tape containing an electrophoresis running buffer. Electroellusion was conducted in the horizontal electrophoresis apparatus using an electric voltage of 120 mV for 90 minutes. After dialyzing, the result of the SDS-PAGE band's protein extraction was ready for the hemagglutination test [10].

#### 2.1.4. Hemagglutination Assay

Hemagglutination assays conducted referred to Finkelstein and Hanne's method. Before protein purification by electroelution, SDS-PAGE should be performed. Then, the sample was carried out at half of the concentration in a microplate. In each well, mouse red blood cell suspended at a level of 0.5% was added in the same volume and shaken using a rotator plate for 1 minute. After that, the plates were incubated at room temperature for 1 hour. The titer was determined by observing the agglutination of red blood cells [10].

#### 2.1.5. Immunization of Mice

The method of immunization referred to a previous study [9]. The vaccine was given orally to mice using sonde four times at a 1-week interval. After one week of the last vaccination, all of the mice were sacrificed according to ethical clearance protocol.

#### 2.1.6. Antihemagglutination Assay

The agglutination inhibition test was carried out and adjusted, according to Jones and Freter (1976). Samples of rat serum from the immunization results were taken as much as 50 *μ*l, and then inserted into well number 1 microplate V in the top row of wells. After that, serial dilutions were carried out in half the dilution starting from well number 2 towards the right up to well number 10, number 11 was empty, and well 12 without serum was only filled with erythrocyte fluid. As for the diluent, PBS solution pH 7.4. Furthermore, in each of the wells, 50 *μ*l of hemagglutinin protein contained two times the highest titer resulting from the hemagglutination reaction. Then the incubation is carried out in a water bath at 37°C for half an hour. After completing incubation time, 50 ml of blood cells of 0.5% mice was wadded in each well. Then, we read the results of the hemagglutination inhibition after incubation at room temperature for 1 hour [10].

### 2.2. The Experimental Laboratory

#### 2.2.1. The Protectivity Test

To measure the level of water excreted by enterocytes into the intestine lumen, we performed the protectivity tests according to our previous study. Twenty mice Balb/c were separated into four groups. The first group was the negative control group. The second group was infected with *S. flexneri*. The third group was immunized before being affected by *S. flexneri*. The last group was the same as the third but infected with *V. cholerae*. After finishing the immunization program, all of the mice within the group were sacrificed. The intestine organ was taken out from the abdomen. We cut the intestine into two parts, 4 cm proximal and 4 cm distal, and then they were ligated. First, second, third, and fourth groups were instilled with 5 × 10^9^ CFU/mL *S. flexneri*. We then observed all of the pieces of cutting intestinal weight for 60 minutes. Every section of the intestine was measured and placed in a 250 cc bottle with 200 ml containing Hank's balanced salt, NaHCO3, and HEPES solution. We put the bottles in a shaker at 37°C for 1 hour with agitation at a rate of 60x per minute. Subsequently, after 15, 30, and 60 minutes, we measured the intestine section's weight gain [8].

#### 2.2.2. Calculation of Bacteria Colonization

After 4 hours of bacterial exposure, we opened the intestine section, and faeces were removed and washed with sterile Phosphate Buffered Saline (PBS). The intestines were cut 10 × 10 mm and homogenized with a Potter homogenizer using sterile PBS with the same volume. We then collected homogenate, planted it on TCBS medium for *V. cholerae,* and then incubated it at 37°C for 18–24 hours. After that, *V. cholerae* was identified from the grown colony and calculated using a colony counter.

(1) The ELISA method was for the examination of s-IgA, *β*-defensin, and IL-17. We measured the level of IL-17 by using an anti-mouse IL-17 A ELISA kit from BioLegend with a standard method from the blood plasma. After that, we collected blood from mice in EDTA tubes and then centrifuged it for 10 minutes. Then, the supernatant is stored at 70°C.

The level of s-IgA and *β* defensin was measured using an anti-mouse s-IgA ELISA kit from Elabscience and an anti-mouse *β*-defensin ELISA kit from MyBioSource standard method from pooled mucous. We cut the intestine at 10 cm in length, and then mucus was collected by scraping from the intestine after removing the entrails. Subsequently, we pooled mucus containing rich s-IgA and *β*-defensin suspended with a ratio of 1 : 1 PBS. The mucus was centrifuged at 6000 rpm for 30 min at 4°C temperature. Then the supernatant is stored at 4°C [9].

(2) The method of Th17 and T-reg cell count was used. The absolute count of Th17 and T-reg cells was assessed using flow cytometry. PBMCs were adjusted to the concentrations of 1 × 106 cells/L and incubated with various antibodies. We used anti-mouse CD4+CD25+Foxp3-PE antibodies (BioLegend, San Diego, CA) for T-reg detection. Besides, we used anti-mouse CD4+IL-17APE antibodies (BioLegend, San Diego, CA) for Th17 detection. Flow cytometer software (BD) was used, and all samples were analyzed using Cell Quest Pro [11].

### 2.3. Data Analysis

All statistical analyses were performed using SPSS. The research was significant when we achieved *p* < 0.05.

## 3. Results

The first part is the immune response of the protectivity.

### 3.1. The First Step

**Figure 1:**
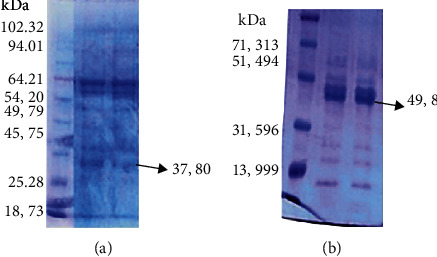
Profile of MW of protein pili subunits *V. cholerae* (a) and *S. flexneri* (b) used SDS-PAGE.

**Figure 2:**
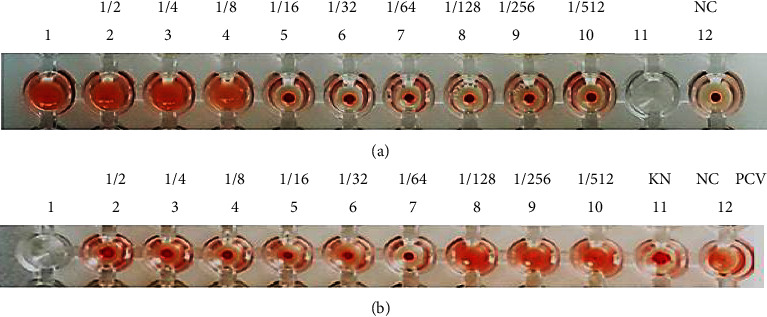
(a) The result of hemagglutination AMV and (b) the result of AMS antibody antihemagglutination AMS inhibited hemagglutination AMV.

The first step was explorative of our research shown in Figures 1, 2(a), and 2(b).

Figure 1 shows MW 37.8 kDa *V. cholerae* and 49.8 kDa *S. flexneri* protein pili subunits. These proteins were AMV and AMS, and for purifying, it did electroelution from five slabs of gel. To evaluate protein concentration per ml of the sample using the nanodrop test, it was noted that there were 0.12 mg/ml and 0.65 mg/ml of *S. flexneri* and *Vibrio cholerae* protein concentration, respectively.

After that, our efforts continued hemagglutination and antihemagglutination assay performed, as shown in Figures 2(a) and 2(b).

We depicted the results in Figure 2(a). Serial twofold dilution of AMS was made from second through twelfth and got the titer 1/8.  Figure 2(b) shows the antihemagglutination assay to know that anti-ADS antibody can inhibit AMV clumping the red blood cells. The titer of the assay shows 1/64. The explorative methods confirmed that we got the antibody anti-AMV shown to inhibit AMS that clumped the red blood cell.

### 3.2. The Second Step

After founding the first step, the result of the explorative research, we performed the second step, namely, the experimental laboratory. The second step is to determine the protectivity's immune response, and we will show the results in the following tables and figures.

**Figure 3:**
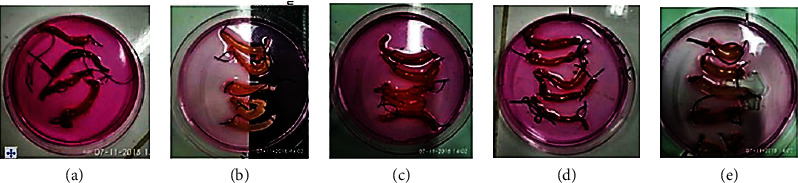
Macroscopic intestinal weight changes of mice vaccinated with *V. cholerae*-infected MLIL method. (a) The first group was the negative control group. (b) The second group we infected with *S. flexneri*. (c) The third group was the same as the second group, but the sample group was infected with *V*. *cholerae*. (d) The fourth group was immunized before being affected by *S. flexneri*. (e) The last group is the same as the fourth group but infected with *V*. *cholerae*.

Comparison of intestinal weight quantitative count postvaccination AMS was depicted in Figure 3.

**Figure 4:**
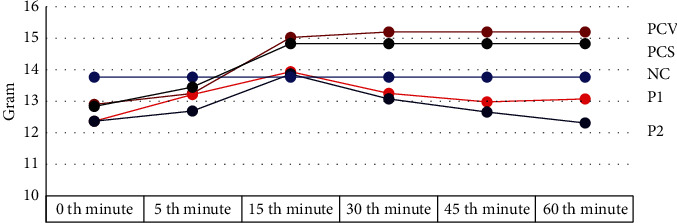
The graph result of weighting every piece of intestinal ligated in the sample.

The weight of every piece of ligated was then observed for 60 minutes. We weighted all samples at 0, 5, 15, 30, 45, and 60 minutes, respectively. The results are shown in Figure 4.

Intestinal weight was measured and performed following 35 days of mice vaccination AMS. Mice were then sacrificed, we cut the intestine into two parts, 4 cm proximal and 4 cm distal, and then these parts were ligated.

Reduced intestinal weights are secondary to *S. flexneri* vaccination. The table and diagram above show intestinal weight changes during 60 minutes of observation after *V. cholerae* installation immediately after MLIL: NC (the first group): negative control; PCV (the second group): *V. cholerae* lively control group; PCS (the third group): *S. flexneri* control group; P1 (the fourth group): *S. flexneri* infected + AMS vaccinated group; P2 (the last group): *V. cholerae*-infected + AMV vaccinated group. Data are mean ± SEM. Statistical analysis was performed based on one-way ANOVA with Tukey's posttest.

**Figure 5:**
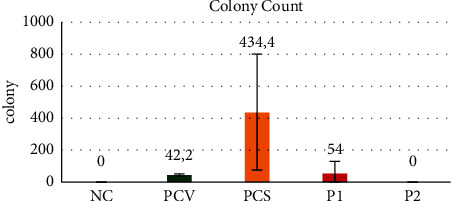
The graph of the number of colony-forming units in the five groups.

We calculated the weight in the second step to count the bacterial colonies, and we pegged Table 1 and Figure 5.

The highest number of colonies we can see is expressed in the *Shigella*-positive control group. However, there is no significant difference between the P1 and negative controls, *Shigella*-positive control, and the P2 group.

In the second part, the others respond to Immun AMS.

We obtained the intestinal secretion. From the intestinal secretion, we can evaluate localized mucosal immunity effectors such as secretory IgA and *β*-defensin. Before that, parenteral vaccination was performed using AMS for 35 days, and then we sacrificed the mice. Figure 3 shows that subunit pili vaccination improves both s-IgA and *β*-defensin levels. S-IgA level secondary to *S. flexneri* vaccination increases compared to the control group and has reached statistical significance. Compared with the control group, there was a 17.62% greater s-IgA level in mice after *S. flexneri* vaccination.

Similarly, another mucosal response indicated by *β*-defensin also significantly elevates in vaccinated mice. The difference between the control and *Shigella*-vaccinated groups is up to 67.86%. These data indicate the presence of immunogenic and protective properties of *S. flexneri* vaccination.

Balb/c mice underwent dissection, and we performed a mucosal secretion analysis. We immediately drew intestinal secretion, and secretory IgA and *β*-defensin levels were measured using ELISA. Data are mean ± SEM. We analyzed statistically based on an independent *t*-test.

IL‐17, Th17, and T‐regulatory systemic adaptive immune responses S. flexneri vaccination given parenterally.

**Figure 6:**
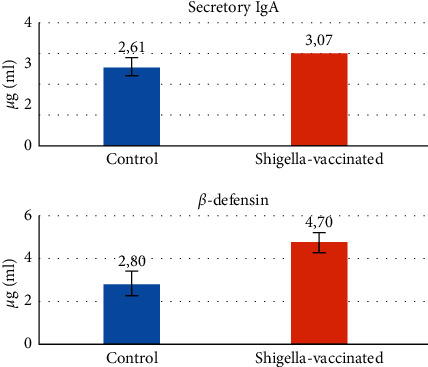
Compared to control, the difference in secretory IgA and *β*-defensin is secondary to *Shigella* vaccination.

To evaluate serum cytokine proinflammatory response post-*S. flexneri* vaccination, IL‐17 A is measured from a blood sample.  Figure 6 illustrates a greater IL-17 response in vaccinated mice compared to the control group. The difference between both groups was 88.03% and statistically significant. Th17 and T-regulatory levels as the cellular response were also measured, and IL-17A, from the data above, noted that S. flexneri vaccination produced a significant difference in both parameters. Compared to the control group, there is elevation of Th17 and T-regulatory, respectively (11.58 ± 1.23 versus 17.59 ± 2.78 and 9.82 ± 2.58 versus 15.22 ± 2.95). T-regulatory and Th17 result is then also interpreted as proportion. The data above showed that there is a domination of Th17 in all study groups. However, there is no significant difference between both groups as Th17 and T-regulatory levels were linear. This result demonstrates the homeostasis of the pro- and anti-inflammatory immune response.

## 4. Discussion

From our previous study using pili cutter and SDS-PAGE, we managed to characterize proteins that lie within pili with molecular weight 7.9 kDa, 11.2 kDa, 27.3 kDa, 49.8 kDa, and 85 kDa in *S. flexneri* and 21.3 kDa, 35.6 kDa, 37.8 kDa, and 50.3 kDa in *Vibrio cholerae*. We took pili of *S. flexneri* and *V. cholerae* with each molecular weight 49.8 kDa and 37.8 kDa and may have the hemagglutinin protein candidate. The selection was based on the previous study that found that an adhesion molecule with a molecular weight of 37.8 kDa in *V. cholerae* [7] and a molecular weight of 49.8 kDa in *S. flexneri* [6] was a hemagglutinin (HA) protein and had the highest titer (Figure 1).

We evaluated the activity of the hemagglutinin protein of *S. flexneri's* pili, hemagglutination (Figure 2). The journals considered that hemagglutinin is one of the critical virulence factors of pathogenic bacteria. Bacteria can perform agglutination and also attach to the erythrocyte cell. The phenomenon can bind to receptors on the host's mucosal cells because of the similarity of both receptors [12]. If the protein has the highest titer, then we use the protein in the antihemagglutination test. We did the antihemagglutination test to show the inhibition of mice erythrocyte agglutination. The results found that *S. flexneri* polyclonal antibodies can inhibit the mice's erythrocyte agglutination. Firstly, we did a hemagglutination test, followed by an antihemagglutinin, and we can conclude that the results show cross inhibition between the polyclonal antibody of AMS with AMV. The serum content antibody shows this expression. The antibody can prevent hemagglutination by binding to the bacterial hemagglutinin protein.

We also investigate what immunology effectors did as vaccination response, either innate arm (*β*-defensin) or adaptive arm of mucosal immunity (secretory Ig A, Th17, its hallmark cytokine IL-17, and T-reg). The effect of 35 days of AMS vaccination conjugated with cholera toxin B on mice demonstrated a significantly high *β*-defensin level (Table 2; Figure 6). *β*-Defensin against a pathogen that binds negatively charged microbial membranes that cause cell death and chemoattraction of immune cells [13]. A high level of *β*-defensin following AMS vaccination consistent with IL-17 result was also elevated significantly. These data concur with the clinical study on humans and mice correlating IL-17 and *β*-defensin from airway epithelial cells. IL-17 proved to be critical for *β*-defensin upregulation against mucocutaneous infections [14, 15]. The CD4+ T cells producing IL-17 as an effector of mucosal immunity reside in intestinal lamina propria [16]. This study showed a high expression of Th17 cells in the spleen of vaccinated mice, so we suspect systemic immune apparatus involvement that acts as an immune response following oral administration of AMS. Th17 has been shown to contribute to the inflammatory response and has a vital role in the host's defence against bacterial and fungal pathogens, especially in the mucosa [17].

**Figure 7:**
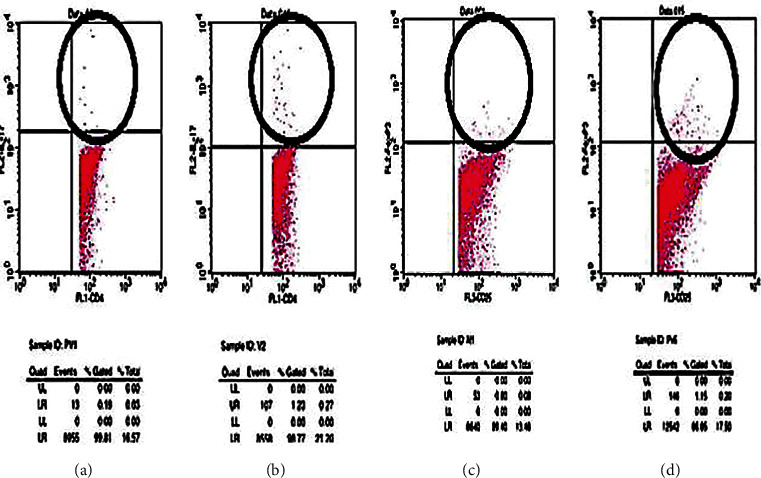
Scatter graph of Th17 proportion (pointed with a black circle) in the control group (a) and *S. flexneri* 49.8 kDa subunit pili vaccinated group (b). Scatter graph of T-regulatory proportion (pointed with black circles) in the control group (c) and *S. flexneri* 49.8 kDa subunit pili vaccinated group (d).

**Figure 8:**
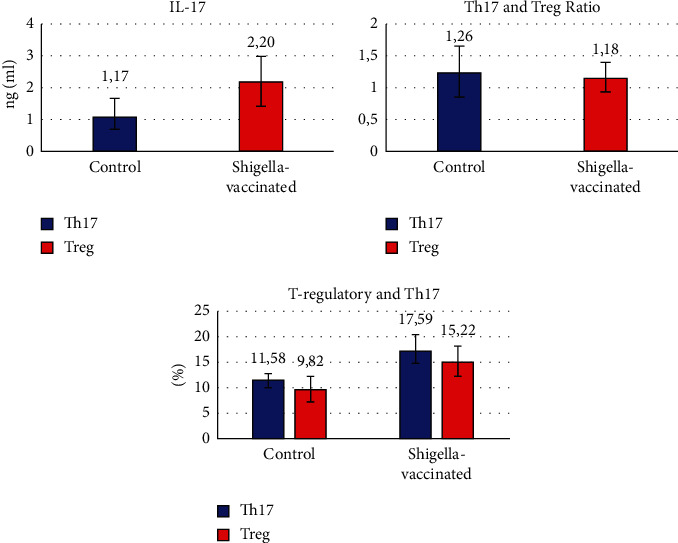
Profile of IL-17, Th17, Th-reg, and ratio Th17 T-reg.

To prevent excessive damage, the tissue-making balances proinflammatory activity in the intestine. So that it does not cause excessive wear to tissues, and anti-inflammatory activity is required. The balance, which is ridden by a subset of other helper T cells, also has TGF-*β*, namely, regulatory T cells [18]. In addition to observing Th17 cells in the splenic, we also observed regulatory T (T-reg) cells characterized by intracellular FoxP3 transcription factor expression. In vaccinated mice, T-reg cell expression was also higher as was found in T-reg in unvaccinated mice. These results demonstrate the function of T-reg in the homeostasis of pro- and anti-inflammatory immune responses (Figure 7).  Figure 8 shows that Il-17 between control with shigella vaccinated no significant difference, but Th17 and Treg ratio between control with shigella vaccinated significally different, and Treg and Th17 between control with shigella vaccinated also significally different.

Additionally, AMS oral vaccination conjugated with CTB also induced a high level of s-IgA as long-term immunity (Table 2 and Figure 6). The intestinal secretion from the control group and the vaccinated group, as measured by ELISA, demonstrated a significant difference. This result indicated mucosal second-line defence after vaccination [19, 20]. S-IgA will prevent the attachment of bacteria and toxins to the epithelial surface [21]. They thus limited pathogen-associated molecular pattern (PAMP) activity to induce an inflammatory response. The selection of CTB as an adjuvant seems to be useful to induce mucosa immunity in line with previous studies using CTB as an adjunct to mucosal immunization [22, 23].

Our previous experiment evaluated by MLIL using AMV shows protection characteristics against *V. cholerae*. We noted a diminished intestinal weight related to lower liquid secretion based on intestinal weight results [7]. We further demonstrated that AMS oral vaccination also promoted protection against *V. cholerae* and *S. flexneri* infection. In addition to this data, we conduct colony counting. We observed inhibition of colony growth of both *V. cholerae* and *S. flexneri* on TCBS and SSA medium from this procedure, respectively. This result further strengthens our hypotheses regarding both organisms' cross-reactivity due to significant intestinal weight and colony reduction after *S. flexneri* and *V. cholerae* vaccinations (Figures 3, 4, and 5 and Tables 1 and 3).

The CD4+ T cells producing IL-17 as an effector of mucosal immunity reside in intestinal lamina propria [16]. We found high expression of Th17 cells in the spleen of vaccinated mice, so we suspect systemic immune apparatus involvement that acts as an immune response following oral administration of AMS. The Th17 has been shown to contribute to the inflammatory response and has an essential role in the host's defence against bacterial and fungal pathogens, especially in the mucosa [17].

## 5. Conclusion

The findings of this experiment prove the existence of a cross-reaction between AMV with AMS. The results described hemagglutinin and antihemagglutinin response, inhibition of colony growth, and diminished intestinal fluid secretion. These findings have strengthened an increase in the mucosal immune effector. This type of response is elicited in both innate and adaptive immune responses following oral administration of AMS. After that, the results of the homologous vaccine candidate immune response between shigella and cholera vaccines still require further research.

## Figures and Tables

**Table 1:** The result of the number of colony-forming units in the five groups.

Groups		Mean ± SD	*p*
NC	Naive group	0 ± 0^b^	0.002
PCV	*Vibrio*-positive control group	42.2 ± 6.61^bc^	
PCS	*Shigella*-positive control group	434.4 ± 364.27^a^	
P1	*Shigella* infection + *Shigella*-vaccinated group	54 ± 73.97^bc^	
P2	*Vibrio* infection + *Shigella*-vaccinated group	0 ± 0^b^	

There are statistical differences in font differences in superscribing, but if there is no statistical difference font that shows no differences in superscribing, and p is the value results of statistical evaluation. The statistical differences with (p≤0.05).

**Table 2:** Compared to control, the difference in secretory IgA and *β*-defensin is secondary to *Shigella* vaccination.

*Secretory IgA*		

Group	Mean ± SD	*p*
Control group	2.61 ± 0.30^b^	0,02
*Shigella*-vaccinated group	3.07 ± 0.11^a^	

*β*-Defensin		

Group	Mean ± SD	*p*
Control group	2.80 ± 0.53^b^	0,000
*Shigella*-vaccinated group	4.70 ± 0.46^a^	

There are statistical differences in font differences in superscribing, but if there is no statistical difference font that shows no differences in superscribing, and p is the value results of statistical evaluation. The statistical differences with (p≤0.05).

**Table 3:** The result of weighting every piece of intestinal ligated in the sample.

NC	PCV	PCS	PI	P2
13,764	12,948	12,872	12,380	12,412
13,764	13,244	13,446	13,214	12,718
13,764	15,044	14,856	13,974	13,878
13,764	15,250	14,848	13,280	13,128
13,764	15,246	14,840	13,020	12,714
13,764	15,242	14,834	13,114	12,354

## Data Availability

The data used to support the findings of this study are available from the corresponding author upon request.

## Abbreviations

AMV: Adhesion molecules of *V. cholerae*
AMS: Adhesion molecules of *S. flexneri*
MLIL: Mice ligated ileal loop
MW: Molecular weight
PAMP: Pathogens-associated molecular pattern
OMP: Outer membrane protein.
